# Pregnancy loss and subsequent risk of prediabetes, diabetes and metabolic syndrome in couples: Tehran lipid and glucose study

**DOI:** 10.1186/s12967-022-03578-2

**Published:** 2022-08-18

**Authors:** Maryam Rahmati, Marzieh Saei Ghare Naz, Fereidoun Azizi, Fahimeh Ramezani Tehrani

**Affiliations:** 1 Reproductive Endocrinology Research Center, Research Institute for Endocrine Sciences, Shahid, Beheshti University of Medical Sciences, Tehran, Iran; 2 Endocrine Research Center, Research Institute for Endocrine Sciences, Shahid Beheshti University of Medical Sciences, Tehran, Iran

**Keywords:** Pregnancy loss, Diabetes, Prediabetes, Metabolic syndrome, Couple

## Abstract

**Background:**

There is limited evidence regarding the impact of pregnancy loss on the subsequent risk of metabolic disorders. We aimed to investigate whether history of pregnancy loss is associated with the subsequent risk of prediabetes (pre-DM), diabetes (DM), and metabolic syndrome (METs) among couples.

**Method:**

In this population-based cohort study, 2765 couples with and without history of pregnancy loss and free of DM, pre-DM, and METs at baseline were included and followed for incidents of DM, pre-DM, and METs by 3-year intervals visits from 1999 to 2018. Detailed data of variables was collected using standard questionnaires, interviews, clinical and laboratory assessments. A modified Poisson regression for binary outcome data with a log link function and robust error variance was used to estimate relative risks (RRs) in couples with and without history of pregnancy loss. Both unadjusted and adjusted models were fitted, and effect measures were calculated.

**Result:**

During a median follow-up of 15 years, females with history of pregnancy loss were experienced more pre-DM (50% vs. 45.5%), DM (28.9% vs. 21.3%), and METs (70% vs. 60.1%) than females without such history. Moreover, history of pregnancy loss increased the risk of METs by 8% among females. The incidence of DM in males with history of pregnancy loss in their spouses was higher than in males without it (28.8% vs. 23.5%). Among males, having a spouse with history of pregnancy loss was positively associated with the risk of pre-DM (RR = 1.12; 95%CI: 1.02, 1.23, p = 0.02); furthermore, they were more prone to the risk of METs than females with a history of pregnancy loss (RR = 1.13; 95%CI: 1.07, 1.20, p < 0.001).

**Conclusion:**

Although pregnancy loss is a female-specific factor, may foreshadow the subsequent METs, our study identified a higher risk of subsequent pre-DM and METs in males with history of pregnancy loss in their spouses. Pregnancy loss could be considered a possible future risk factor for metabolic disorders in couples.

## Introduction

Metabolic syndrome (METs), diabetes mellitus (DM), and prediabetes (pre-DM) are the leading health problems around the world [1–3] and could lead to poor cardiovascular outcomes [4–6]. Despite the introduction of numerous established risk factors for DM and METs (such as physical inactivity, smoking, and unhealthy diet) [7, 8], there are still unknown factors that contribute to developing metabolic disorders in both males and females. Recently, gender-specific differences between females and males in terms of cardio-metabolic risk factors have been suggested [9, 10]. For example, pregnancy complications are known as unique risk factors for cardio-metabolic disorders in women [11].

It is well established that each pregnancy poses a diabetogenic effect on maternal metabolism [12]. Indeed, normal pregnancy is cardio-metabolic stress, and any pregnancy complication might be preceded by a metabolic abnormality [13, 14]. Pregnancy loss, both miscarriage and stillbirth, is a common pregnancy complication [15]. Several studies have shown that history of pregnancy loss (PL) in women is linked to subsequent chronic conditions like cardiovascular disease and renal disease [16–19]; however, there are limited and inconsistent reports of the association between pregnancy loss and the risk of metabolic disorders in mothers later in life [18, 20, 21].

The spousal concordance of cardio-metabolic risk factors received more attention in recent literature [22, 23]. A cohort study among middle-aged adults has demonstrated that males whose wives have a history of CVD were more prone to CVD [24]. Another study supported the concordance of glycaemic and cardio-metabolic parameters among females with a history of gestational diabetes and their spouses [25]. Moreover, several studies revealed the increased risk of developing metabolic disorders in spouses of women who experience pregnancy complications such as hyperglycemia during pregnancy, gestational diabetes, and gestational hypertension [26–28].

The exact mechanisms related to the adverse pregnancy outcomes and disease risk in couples later in life remain incompletely understood. It has been proposed that rather than biological factors related to pregnancy, some non-biological factors contribute to an increased risk of chronic disease in males and females [29]. Concordance regarding lifestyle factors may lead to the development of the same chronic disease in couples [30]. It seems that behaviors and lifestyle which implemented years after complicated pregnancy might affect the risk of metabolic disorders among couples.

Pregnancy loss is among the most common complication of pregnancy and might be a stressor event for both parents [31]; as a result, it acts as a trigger for metabolic abnormality [32]. A limited number of studies have investigated the risk of diabetes in women with a history of pregnancy loss [18, 20, 21], but to our knowledge, there is still no study addressing the role of pregnancy loss on the risk of METs and pre-DM; additionally, the adverse effect of pregnancy loss on spousal risk of metabolic disorders has not been investigated yet.

In the present study, we aimed to investigate the risk of pre-DM, DM, and METs in couples with pregnancy loss history in a population-based cohort study with, on average, 15 years of follow-up.

## Method

This study has been undertaken using data from an ongoing population-based longitudinal Tehran Lipid and Glucose Study. The protocol of TLGS was developed according to the World Health Organization approach for surveillance of risk factors for non-communicable diseases [33]. The baseline phase of this ongoing cohort was conducted between 1999 and 2001, and follow-up phases were performed at 3-year intervals. The population of this study was selected from residents of district number 13 of Tehran (a representative sample of Tehran), the capital of Iran. Detailed information on the study design and the rationale behind the methodology has been addressed elsewhere [34].

### Study population

In the present study, among a total of 20,145 subjects in TLGS, there were 3,650 matched couples. Regarding the female participants, we chose individuals who met the following criteria: married females aged > 18 at baseline, females whose spouses had complete records of variables, and females without DM, pre-DM, and METs at baseline and before exposure to pregnancy loss. Also, the inclusion criteria for males include married males aged > 18 at baseline, males whose spouses had complete records of variables, and males without DM, pre-DM, and METs at baseline and before exposure to pregnancy loss in their spouses.

**Fig. 1:**
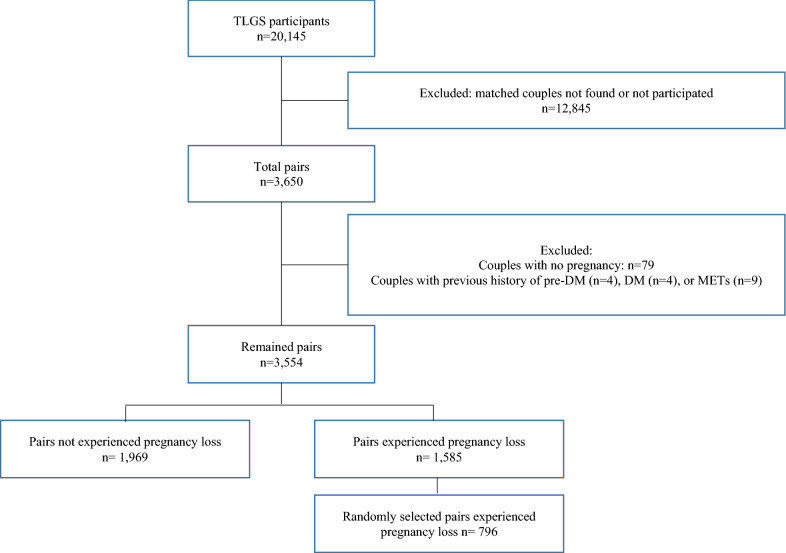
Flowchart of the study

After exclusion of those with history of pre-DM (n = 4), DM (n = 4), or METs (n = 9), and couples with no pregnancy (n = 79), finally, 3,554 couples remained eligible for the present analysis. Of the remaining participants, 1585 have history of pregnancy loss. In order for the percentage of women with history of pregnancy loss to represent the community (around 25% to 30%), we selected a random sample of these women (n = 796). Eventually, 2765 couples, including 1969 women who had never experienced pregnancy loss and 796 women who had experienced at least one pregnancy loss over the follow-up, remained in this study (Fig. 1). Couples were included from the first (n = 2362), second (n = 381), and third (n = 22) phases and followed until the end of the study (20 March 2018).

### Measures

Trained staff and physicians studied the participants of this study according to the standard protocol of TLGS. Also, a standard and validated questionnaire was used to gather demographic and medical history and variables [34]. Anthropometric, laboratory, and clinical assessments were performed based on the TLGS measurement protocol. All blood analyses were carried out at the TLGS research laboratory. Details of measurement of systolic and diastolic blood pressure, anthropometric parameters, and laboratory measurements, including fasting blood glucose (FBS) levels, lipid profile triglyceride (TG), low-density lipoprotein cholesterol (LDL), high-density lipoprotein cholesterol (HDL), and total cholesterol (TC), have been reported previously [34].

Overall, variables including age, marital status, smoking status, education level, physical activity, number of gravidity or parity, and medical and drug history were obtained through self-reported questionnaires. The details of a female’s obstetric history, including pregnancy outcomes, were collected through a review of relevant medical documents and face-to-face interviews. Other variables which measured in this study were collected from both females and males using standard questionnaires, interviews, clinical and laboratory assessments.

In this study’s exposure variable, pregnancy loss was defined as history of any type of abortion or miscarriage, or stillbirth [15, 35]. Outcomes interest variables were as DM, METs, and pre-DM. Detailed information about these outcomes has been published elsewhere [36].

The Modifiable Activity Questionnaire (MAQ) [37], which was reliable and validated in the Iranian population, was used for assessing physical activity. This questionnaire measures the physical activities related to leisure time, household, and occupational activities. The metabolic equivalent (MET) was calculated based on min/week. 1500 min/week and appropriate physical activity defined as MET ≥ 600 min/week.

### Statistical analysis

Continuous variables were checked for normality using the Shapiro–Wilk test; those with normal distribution were expressed as mean (standard deviation), and non-normal distributed variables were expressed as median (interquartile range). Categorical variables were expressed as percentages. Characteristics of participants were compared between the pregnancy loss categories by applying the independent t-test or Pearson’s Chi-squared test for continuous and categorical data, respectively. The Mann–Whitney U test was applied to compare variables with skewed distribution.

For this study, we do a post hoc analysis, which involves looking at the data after a study has been concluded and trying to find patterns that were not the primary objectives of the study. TLGS was initiated in 1999 to investigate non-communicable disease (NCD) and its associated risk factors or determinants among a representative family-based population of Tehran, the capital of Iran; however, in this study, we aimed to discover the impact of pregnancy loss on the subsequent risk of metabolic disorders among couples.

A modified Poisson regression for binary outcome data with a log link function and robust error variance was used to estimate relative risks (RRs) and 95% confidence intervals (CIs) for the associations between pregnancy loss and incidence of pre-DM, DM, and METs in males and females over the follow-up [38]. We considered three models for this analysis; model 1: unadjusted model, model 2: age-BMI adjusted model, and model 3 was adjusted for age, WHtR, BMI, education, parity, number of pregnancy loss, SBP, FBS, TG, TC, LDL, and family history of diabetes. Adjusting variables were determined based on the significant differences between those participants who experienced pregnancy loss and those who did not. Moreover, to adjust the results for matching cases and achieve a robust variance, we considered the couples as the cluster observations in the model. Finally, the plots of the relative risks were depicted for three outcomes and sex groups by pregnancy loss status. Statistical analysis was performed using the software package STATA (version 13; STATA Inc., College station, TX, USA); the significance level was set at P < 0.05.

## Result

In the present study, 2765 couples were included. Among those pairs, 1969 (71.2%) had no pregnancy loss, and 796 (28.8%) experienced at least one pregnancy loss. Among couples with history of pregnancy loss, those with only abortion history were 618, only stillbirth history were 42, and those who experienced both types were 136 couples.

The total median (IQR) follow-up time was 15 (10–16) years which was 14 (8–16) years, and 15 (11–16) years for males and females, respectively.

Tables 1 and 2 summarize the characteristics of participants at the baseline and last follow-up for females and males, respectively. The characteristics are categorized by the pregnancy loss status. According to the baseline part of Table 1, the mean entry age was higher in both males and females in group with history of pregnancy loss (49.0 (11.8) for males and 42 (11.0) for females) compared to no group without history of pregnancy loss (44.5 (11.4) for males and 37.9 (10.4) for females) (p < 0.001). Couples with history of pregnancy loss also had the highest waist to hip ratio (males: mean (SD): 0.54(0.06) vs. 0.53(0.06)) and females: (mean (SD): 0.57(0.08) vs. 0.56 (0.08)) SBP (males: mean (SD): 122.5(19.9) vs. 119.5(17.6)) and females: (mean (SD): 118.6(19.3) vs. 115.1 (16.8)), FBS (males: mean (SD): 5.6(1.9) vs. 5.5(1.7)) and females: (mean (SD): 5.4(1.8) vs. 5.3 (1.6)), TC (males: mean (SD): 5.4(1.1) vs. 5.3(1.0)) and females: (mean (SD): 5.5(1.2) vs. 5.3 (1.2)), and lower educational status (males: number (percentage): 352(44.3) vs. 1019(51.8) and females: number (percentage): 296(37.3) vs. 911 (46.3)). According to pregnancy history, the median of number of pregnancy losses in couples with pregnancy loss was 1 (IQR: 1, 2) (Table 1).

**Table 1:** Baseline characteristics of the females and males with no pregnancy loss and with pregnancy loss

Characteristics	Females			Males		
	Not experienced pregnancy loss N = 1969	Experienced pregnancy loss N = 796	p-value^d^	Their spouses not experienced pregnancy loss N = 1969	Their spouses experienced pregnancy loss N = 796	p-value^d^
Age^a^ (years)	37.9 (10.4)	42.0 (11.0)	< 0.001	44.5 (11.4)	49.0 (11.8)	< 0.001
Smoking status^c^ (current + past)	86 (4.4)	32 (4.0)	0.7	903 (46.2)	380 (48.0)	0.4
WHtR^a^	0.56 (0.08)	0.57 (0.08)	< 0.001	0.53(0.06)	0.54(0.06)	0.003
BMI ^a^ (kg/m^2^)	27.8 (4.7)	28.6 (4.7)	0.004	26.3 (4.0)	26.4 (3.8)	0.4
Appropriate physical activity^c^	610 (31.0)	262 (32.9)	0.3	550 (28.2)	236 (29.8)	0.4
SBP^a^ (mmHg)	115.1 (16.8)	118.6 (19.3)	< 0.001	119.5 (17.6)	122.5 (19.9)	< 0.001
DBP^a^ (mmHg)	76.3 (10.2)	78.1 (10.9)	< 0.001	78.1 (10.7)	79.0 (11.5)	0.06
FBS^a^ (mmol/l)	5.3 (1.6)	5.4 (1.8)	0.04	5.5 (1.7)	5.6 (1.9)	0.03
TG^b^ (mmol/l)	1.4 (1.0–2.1)	1.7 (1.1–2.4)	< 0.001	1.8 (1.3–2.6)	1.9 (1.3–2.7)	0.2
TC^a^ (mmol/l)	5.3 (1.2)	5.5 (1.2)	0.002	5.3 (1.0)	5.4 (1.1)	0.03
HDL^a^ (mmol/l)	1.1 (0.3)	1.1 (0.3)	0.9	1.0 (0.2)	1.0 (0.2)	0.9
LDL^a^ (mmol/l)	3.4 (1.0)	3.5 (1.0)	0.02	3.4 (0.9)	3.4 (0.9)	0.2
Education^c^ (diploma and upper)	911 (46.3)	296 (37.3)	< 0.001	1,019 (51.8)	352 (44.3)	< 0.001
Family history of DM^c^	857 (43.5)	346 (43.5)	0.9	758 (38.5)	275 (34.5)	0.06
Gravidity^a^	1.0 (1.6)	1.5 (2.3)	< 0.001	1.0 (1.6)	1.5 (2.3)	< 0.001
Parity^a^	1.0 (1.6)	1.2 (1.9)	0.001	1.0 (1.6)	1.2 (1.9)	0.001

*WHtR* Waist-to-Height Ratio, *BMI* body mass index, *FBS* fasting blood glucose, *pre-DM* prediabetes, *DM* diabetes mellitus, *METs* metabolic syndrome, *SBP* systolic blood pressure, *DBP* diastolic blood pressure, *TG* triglyceride, *LDL* low-density lipoprotein cholestero, *HDL* high-density lipoprotein cholestero, *TC* total cholesterol

^a^Values are presented as mean (SD), ^b^values are expressed as median (Inter Quartile Range), ^c^data shown as number (percentage). ^d^Significant differences (P-value < 0.05), analyzed using independent samples t-test for superscripts a, Mann–Whitney U test for superscripts b and Pearson’s test for superscripts c

**Table 2:** Last follow-up characteristics of the females and males with no pregnancy loss and with pregnancy loss

Characteristics	Females			Males		
	Not experienced pregnancy loss N = 1969	Experienced pregnancy loss N = 796	p-value^d^	Their spouses not experienced pregnancy loss N = 1969	Their spouses experienced pregnancy loss N = 796	p-value^d^
Age ^a^ (years)	50.3 (11.9)	55.0 (12.0)	< 0.001	55.9 (12.5)	60.6 (12.5)	< 0.001
Smoking status ^c^(current + past)	77 (3.9)	36 (4.5)	0.4	919 (47.0)	369 (46.5)	0.8
WHtR ^a^	0.61 (0.08)	0.63 (0.08)	< 0.001	0.56 (0.07)	0.57 (0.06)	0.04
BMI ^a^ (kg/m^2^)	29.8 (5.0)	30.5 (5.2)	0.004	27.1 (4.4)	27.1 (4.1)	0.9
Appropriate physical activity ^c^	576 (29.3)	244 (30.6)	0.5	805 (41.2)	339 (42.7)	0.5
SBP ^a^ (mmHg)	116.9 (18.9)	120.0(19.4)	< 0.001	122.7 (18.6)	125.8 (20.5)	< 0.001
DBP ^a^ (mmHg)	76.6 (10.1)	77.4 (10.4)	0.06	79.1 (10.5)	78.9 (11.7)	0.7
FBS ^a^ (mmol/l)	5.6 (1.9)	5.9 (2.1)	0.004	6.0 (2.2)	6.1 (2.2)	0.2
TG ^b^ (mmol/l)	1.4 (1.0–2.0)	1.5 (1.1–2.0)	0.1	1.6 (1.1–2.2)	1.6 (1.1–2.2)	0.5
TC ^a^ (mmol/l)	5.1 (1.1)	5.1 (1.1)	0.9	4.9 (1.0)	4.9 (1.1)	0.7
HDL ^a^ (mmol/l)	1.3 (0.3)	1.3 (0.3)	0.6	1.1 (0.2)	1.1 (0.3)	0.5
LDL ^a^ (mmol/l)	3.1 (1.0)	3.0 (1.0)	0.5	3.0 (0.9)	3.0 (1.0)	0.4
Gravidity ^a^	3.0 (1.7)	5.2 (2.6)	< 0.001	3.0 (1.7)	5.2 (2.6)	< 0.001
Parity ^a^	3.0 (1.7)	3.8 (2.2)	< 0.001	3.0 (1.7)	3.8 (2.2)	< 0.001
Incidence pre-DM ^c^	895 (45.5)	398 (50.0)	0.03	1,011 (51.3)	429 (53.9)	0.2
Incidence DM ^c^	419 (21.3)	230 (28.9)	< 0.001	463 (23.5)	229 (28.8)	0.004
Incidence METs ^c^	1,184 (60.1)	557 (70.0)	< 0.001	1,434 (72.8)	605 (76.0)	0.09

*WHtR* Waist-to-Height Ratio, *BMI* body mass index, *FBS* fasting blood glucose, *pre-DM* prediabetes, *DM* diabetes mellitus, *METs* metabolic syndrome, *SBP* systolic blood pressure, *DBP* diastolic blood pressure, *TG* triglyceride, *LDL* low-density lipoprotein cholestero, *HDL* high-density lipoprotein cholestero, *TC* total cholesterol

^a^Values are presented as mean (SD), ^b^values are expressed as median (Inter Quartile Range), ^c^data shown as number (percentage). ^d^Significant differences (P-value < 0.05), analyzed using independent samples t-test for superscripts a, Mann–Whitney U test for superscripts b and Pearson’s test for superscripts c

The incidence of outcome variables pre-DM, DM and METs in females with history of pregnancy loss increases compared to females with no pregnancy loss (50% vs. 45.5% for pre-DM (p = 0.03), 28.9% vs. 21.3% for DM (p < 0.001) and 70% vs. 60.1% for METs (p < 0.001)). Moreover, there was a significantly higher incidence of DM outcome in males with history of pregnancy loss in their spouses compared to males with no history of pregnancy loss in their spouses (28.8% vs. 23.5% (p = 0.004)) (Table 2).

Table 3 shows the unadjusted and adjusted relative risks of pre-DM, DM, and METs based on poisson regression models when the effect of sex, pregnancy loss, and the interaction term of these two are in the model. Model 1 (unadjusted model) reveals that having pregnancy loss history was associated with 22% higher risk of DM in males (RR = 1.22; 95%CI: (1.07, 1.40), p = 0.003) (Table 2); however, this association was disappeared after adjusting variables (model 2 & 3). Furthermore, Model 3 shows overall males experienced higher risk of pre-DM (RR = 1.14; 95%CI: (1.06, 1.22), p < 0.001), DM (RR = 1.13; 95%CI: (1.01, 1.28), p = 0.04), and METs (RR = 1.22; 95%CI: (1.16, 1.27), p < 0.001) compared to females.

**Table 3:** Poisson regression model analysis for pre-DM, DM, and METs outcomes in relation to pregnancy loss

Variables		Model 1			Model 2			Model 3		
		RR	95% CI	p-value	RR	95% CI	p-value	RR	95% CI	p-value
Pre-DM										
Sex										
	Male/Female	1.14	1.06,1.22	< 0.001	1.12	1.05,1.20	< 0.001	1.14	1.06,1.22	< 0.001
Pregnancy loss										
	Yes/No	1.10	1.01,1.20	0.03	1.05	0.97,1.14	0.2	1.07	0.97,1.18	0.2
Sex * Pregnancy loss										
	Male* Yes	1.05	0.97,1.13	0. 2	1.01	0.94,1.10	0.7	1.04	0.95,1.15	0.3
	Male*No	Reference								
DM										
Sex										
	Male/Female	1.10	0.98,1.23	0.08	1.03	0.92,1.16	0.6	1.13	1.01,1.28	0.04
Pregnancy loss										
	Yes/No	1.36	1.18,1.56	< 0.001	1.11	0.97,1.27	0.1	1.14	0.97,1.32	0.09
Sex * Pregnancy loss										
	Male* Yes	1.22	1.07,1.40	0.003	1.05	0.92,1.20	0.4	1.03	0.89,1.20	0.7
	Male*No	Reference								
METs										
Sex										
	Male/Female	1.21	1.16,1.26	< 0.001	1.24	1.19,1.29	< 0.001	1.22	1.16,1.27	< 0.001
Pregnancy loss										
	Yes/No	1.16	1.10,1.23	< 0.001	1.07	1.01,1.12	0.02	1.08	1.02,1.14	0.01
Sex * Pregnancy loss										
	Male* Yes	1.04	0.99,1.09	0.07	0.98	0.94,1.03	0.5	1.00	0.95,1.06	0.9
	Male*No	Reference								

*WHtR* Waist-to-Height Ratio, *BMI* body mass index, *FBS* fasting blood glucose, *pre-DM* prediabetes, *DM* diabetes mellitus, *METs* metabolic syndrome, *SBP* systolic blood pressure, *DBP* diastolic blood pressure, *TG* triglyceride, *LDL* low-density lipoprotein cholestero, *TC* total cholesterol

Only includes Sex, pregnancy loss status and interaction of them. Adjusted for age and BMI..Adjusted for age, WHtR, BMI, education, parity, number of pregnancy losses, SBP, FBS, TG, TC, LDL, and family history of DM

**Fig. 2:**
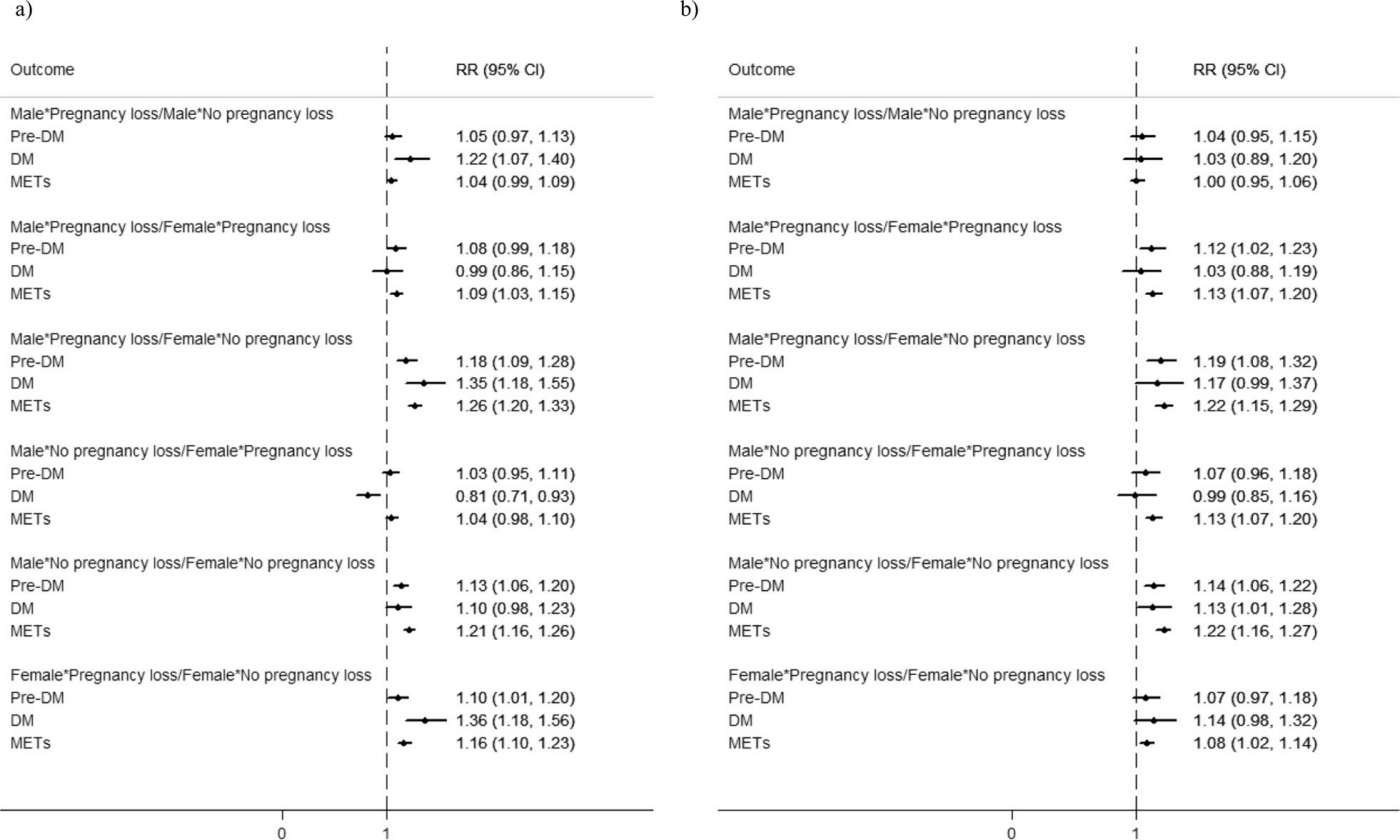
Unadjusted (**a**) and adjusted (**b**) Relative Risks and 95% CIs for pre-DM, DM, and METs outcomes comparing couples with and without history of pregnancy loss. RRs are adjusted for age, WHtR, BMI, education, parity, number of pregnancy losses, family history of DM, SBP, FBS, TG, TC, and LDL. WHtR: Waist-to-Height Ratio; BMI: body mass index; FBS: fasting blood glucose; pre-DM: prediabetes; DM: diabetes mellitus; METs: metabolic syndrome SBP; systolic blood pressure; DBP: Diastolic blood pressure; TG: triglyceride; LDL: low-density lipoprotein cholesterol; TC: total

**Fig. 3:**
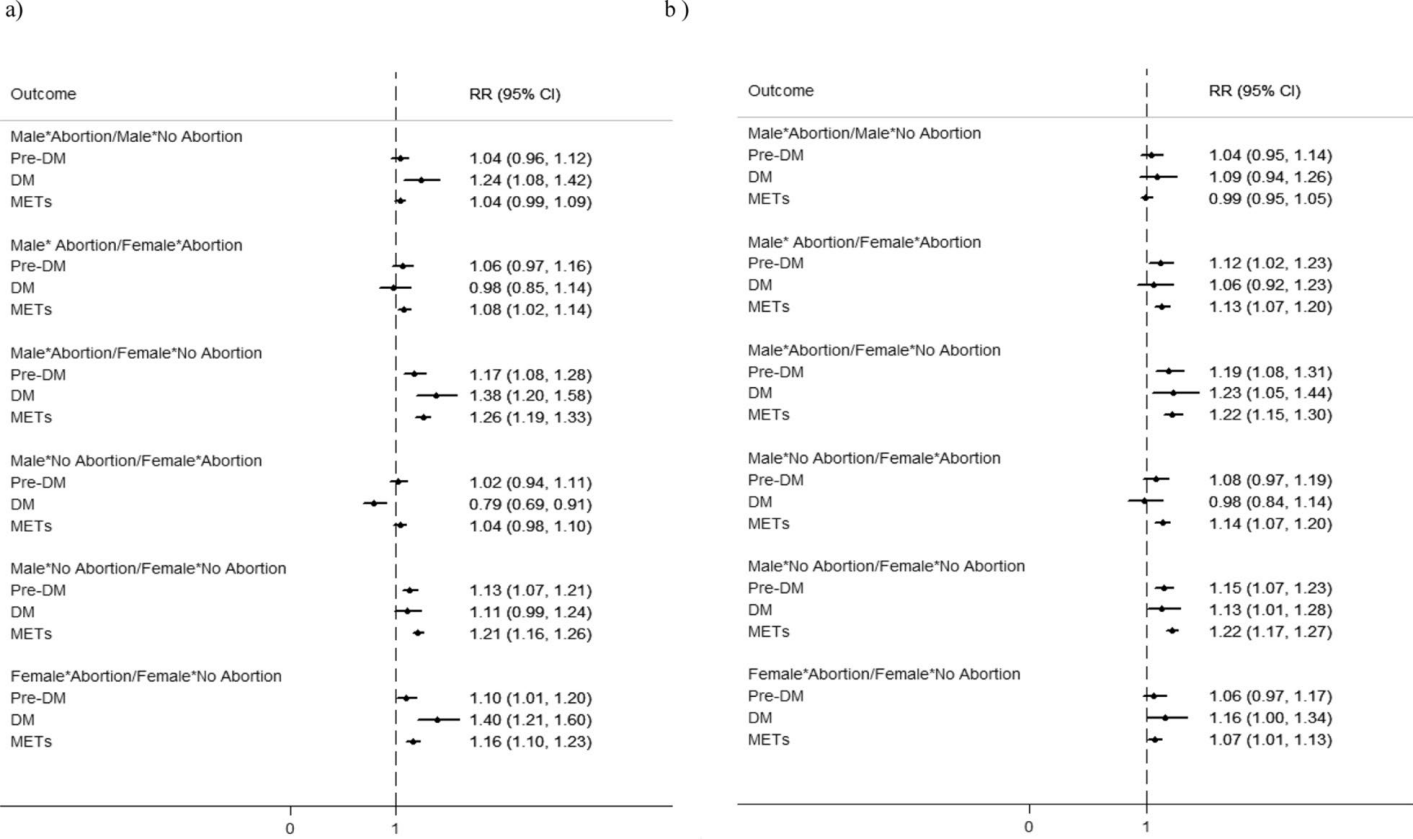
Unadjusted (**a**) and adjusted (**b**) Relative Risks and 95% CIs for pre-DM, DM, and METs outcomes comparing couples with and without history of abortion. RRs are adjusted for age, WHtR, BMI, education, parity, number of pregnancy losses, family history of DM, SBP, FBS, TG, TC, and LDL. WHtR: Waist-to-Height Ratio; BMI: body mass index; FBS: fasting blood glucose; pre-DM: prediabetes; DM: diabetes mellitus; METs: metabolic syndrome SBP; systolic blood pressure; DBP: Diastolic blood pressure; TG: triglyceride; LDL: low-density lipoprotein cholesterol; TC: total

**Fig. 4:**
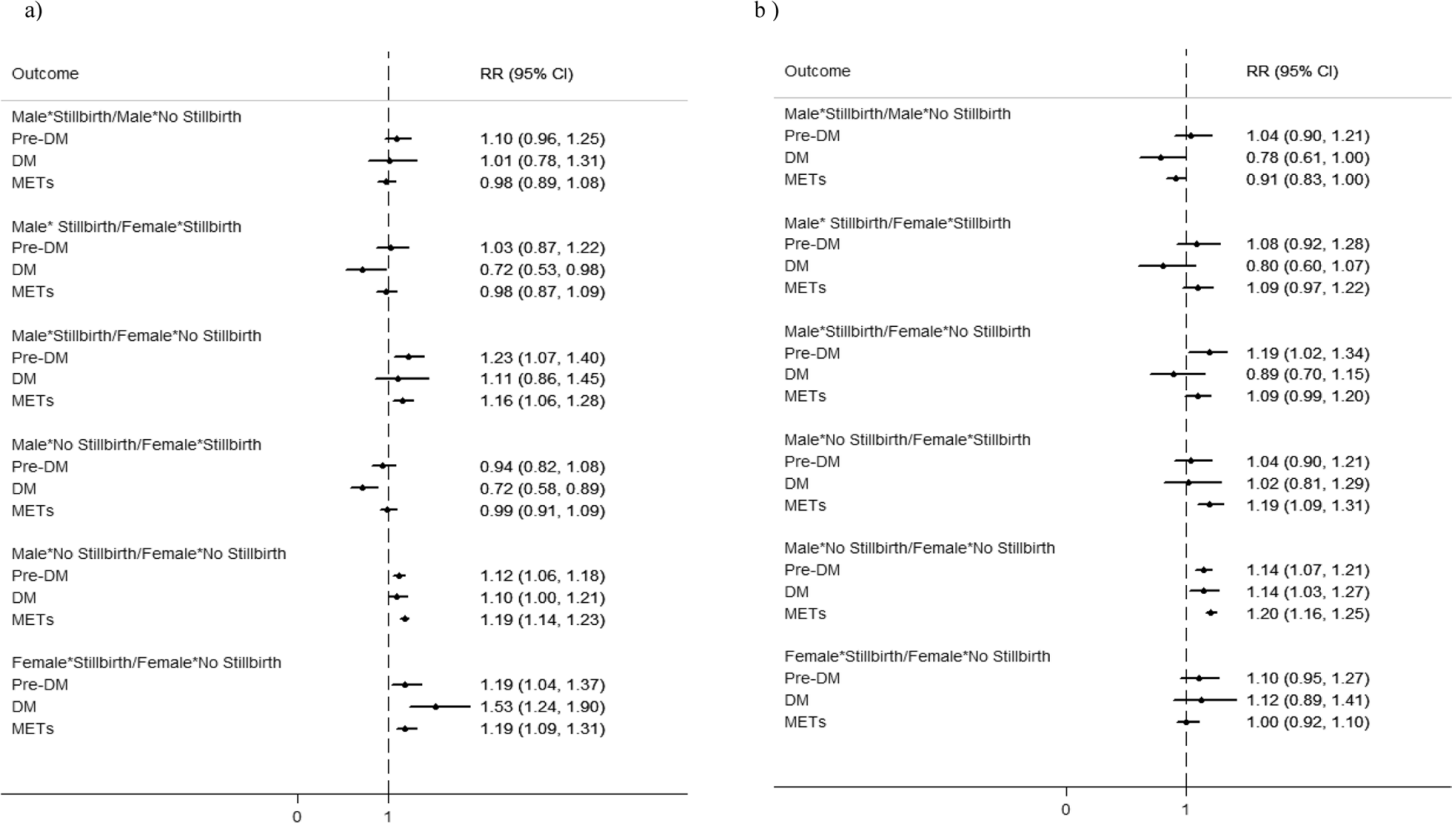
Unadjusted (**a**) and adjusted (**b**) Relative Risks and 95% CIs for pre-DM, DM, and METs outcomes comparing couples with and without history of stillbirth. RRs are adjusted for age, WHtR, BMI, education, parity, number of pregnancy losses, family history of DM, SBP, FBS, TG, TC, and LDL. WHtR: Waist-to-Height Ratio; BMI: body mass index; FBS: fasting blood glucose; pre-DM: prediabetes; DM: diabetes mellitus; METs: metabolic syndrome SBP; systolic blood pressure; DBP: Diastolic blood pressure; TG: triglyceride; LDL: low-density lipoprotein cholesterol; TC: total

**Fig. 5:**
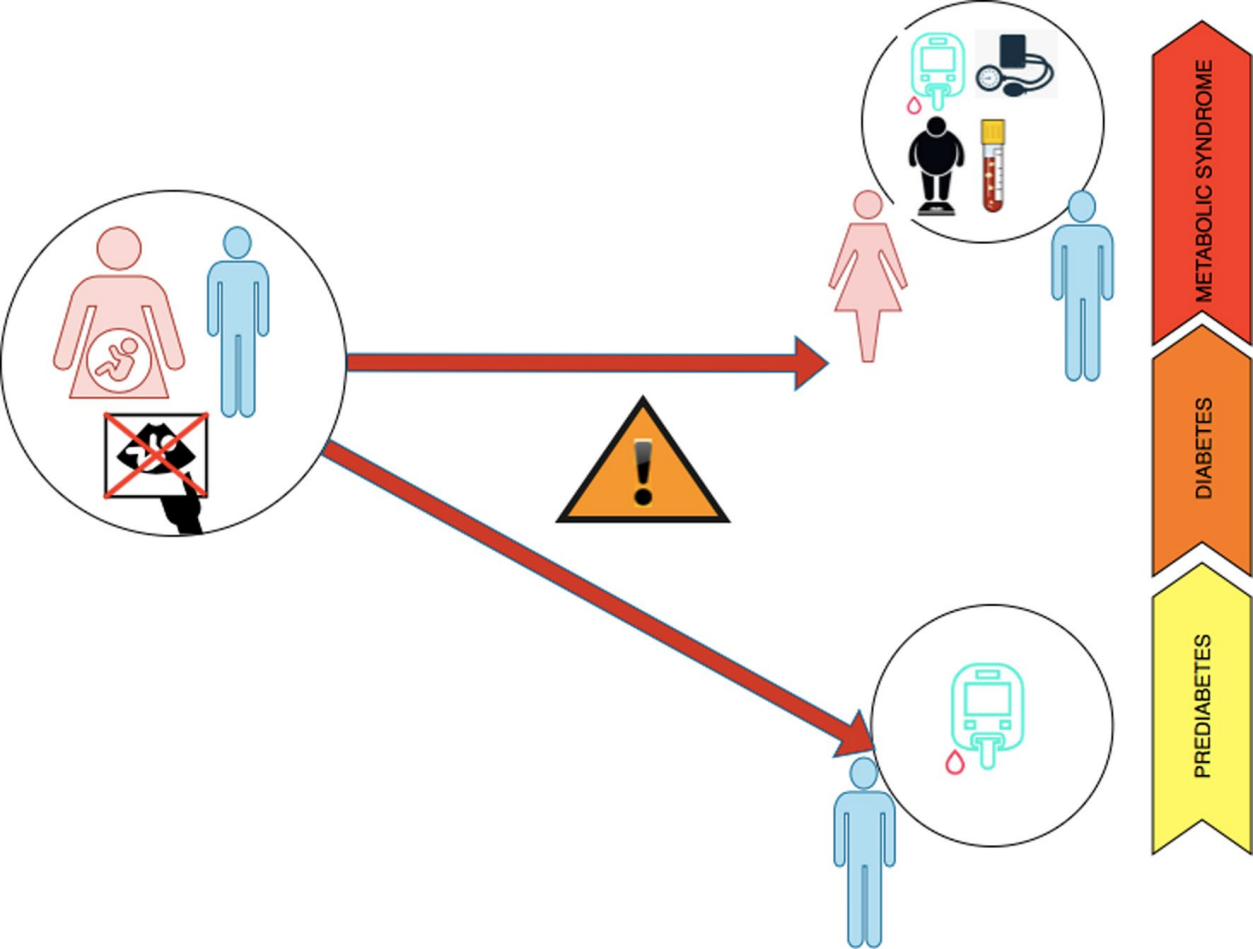
Overall findings of study

Figure 2 represents unadjusted and adjusted relative risks and corresponding 95% confidence intervals of metabolic disorders based on the interaction term sex*pregnancy loss status. In couples with history of pregnancy loss, males were more prone to the risk of pre-DM (RR = 1.12; 95%CI: (1.02,1.23), p = 0.02), and METs (RR = 1.13; 95%CI: (1.07, 1.20), p < 0.001) than females, however, males with history of pregnancy loss revealed no higher risk of metabolic disorders compared to the males without it. Moreover, females with history of pregnancy loss increased the risk of METs by 8% than females without history of pregnancy loss (RR = 1.08; 95%CI: (1.02, 1.14), p = 0.01) (Fig. 2). Additionally, the interaction effects of each type of pregnancy loss (abortion or stillbirth) with sex in relation to the risk of metabolic disorders were presented in Figs. 3 and 4. In couples with history of abortion, males demonstrated higher risk of pre-DM (RR = 1.12; 95%CI: (1.02, 1.23), p = 0.02) and METs (RR = 1.13; 95%CI: (1.07, 1.20), p < 0.001) compared to their spouses. However, couples with a history of stillbirth did not show such an association. Besides, among females, those who had a history abortion were 7% more likely for METs risk (RR = 1.07; 95% CI: (1.01, 1.13); p = 0.02), while females with history of stillbirth had no higher risk of METs compared to females without history of stillbirth. Figure 5 shows cartoon representation of results.

## Discussion

This population-based cohort study was conducted to determine whether couples with a history of pregnancy loss are at an elevated risk of pre-DM, DM, and METs in the long term. The main findings of this study were that males with history of pregnancy loss in their spouses were at increased risk of pre-DM and METs compared to females after adjustment for confounders, while for DM, no significant association was noticed. Moreover, females with history of pregnancy loss just experienced an elevated risk of METs compared with females without such history.

Today, the growing epidemic of METs and DM can be observed worldwide [39]; these disorders are considered as two main major risk factors for CVD [40, 41]. Pregnancy is considered as a potential risk factor for further cardio-metabolic events due to some physiological adaptations that occur during pregnancy [13, 14]. This adverse effect is exaggerated by pregnancy complications including gestational diabetes, preterm delivery, and pregnancy-induced hypertension [42]. This complication not only increases the probability of developing CVD, DM, and HTN in females, but also increases the cardio-metabolic disturbances among their spouses [27, 28]; the exact mechanisms related to this concordance in couples later in life remain incompletely understood. It is assumed that rather than biological factors related to pregnancy, some non-biological factors contribute to an increased risk of chronic disease in males and females [43, 44]. Prior studies demonstrate that women with a history of pregnancy loss are at an increased risk of DM, HTN, and hypercholesterolemia [18, 45]. Furthermore, while pregnancy loss increases the probability of developing CVD risk factors in females [18, 45], by our knowledge, its adverse effect on their spouse’s cardio-metabolic situation has not been reported yet. We found that in addition to females, DM and METs were occurred more often in males with history of pregnancy loss in their spouses; even these males were more prone to the risk of METs than females with history of pregnancy loss.

Among females who participated in the present study, while having a history of at least one pregnancy loss increased the risk of various adverse metabolic disorders (pre-DM, DM, and METs); however, after adjustment for confounders, this association just remained significant for METs. There is limited study in terms of determining the subsequent risk of DM in women with history of pregnancy loss [18, 21, 46] and the subsequent risk of pre-DM and METs in women with history of pregnancy loss had not been reported before, by our knowledge. In line with our study, a cohort study (the Women’s Health Initiative) demonstrated that history of pregnancy loss was associated with a higher rate of DM [18]. It has been shown in another large population-based study that having history of pregnancy loss increases the risk of female CVD [46]. Moreover, a Danish nationwide case–control study among 24,774 women with DM and 247,740 controls revealed that women with a history of pregnancy loss are at increased risk of DM [21]. By contrast, Kharazmi et al. in a prospective cohort study, found that history of abortion and stillbirth was not significantly associated with the risk of DM in women [20]. When we evaluated the possible excessive risk of metabolic disorders in terms of abortion and stillbirth separately, we found that having history of abortion was associated with an increased risk of METs among females. However, couples with history of stillbirth were not more prone to metabolic disorders; however it may be due to the lack of adequate number of stillbirth in present study. Another studies which proposed that any form of pregnancy loss (including stillbirth and miscarriage) may increase women’s future risk of cardio-metabolic disorders [18, 42, 45, 47].

Moreover, our study showed that males with a history of pregnancy loss or abortion in their spouses were more likely to experience METs and per-DM than females. This may be explained by the impact of the paternal metabolic conditions on pregnancy loss. Kasman et al. reported that in men with increasing components of METs in the preconception period, the risk of pregnancy loss was significantly increased [48]. As a result, it is assumed that those males with history of pregnancy loss in their spouses may have a greater baseline risk of metabolic disorders. Moreover, a systematic review revealed that following a pregnancy loss, males might be faced with double-disenfranchised grief [49]. Indeed, lack of support and facing diverse challenges due to pregnancy loss could manifold the disenfranchised grief for males [50]. These men might be at risk for psychological disorders which might share a common pathway with metabolic disorders [32, 51, 52]. Indeed, it is evident that gender differences in sex hormones, energy balance, and body composition may partly explain the susceptibility of males to metabolic disorders [53, 54]. A recent review shows that due to biological sex differences, men are more likely to develop DM in middle age groups [55]. Additionally, spouses are concordant in lifestyle habits [56]. So spouses who have an unhealthy lifestyle are more likely to develop cardiovascular risk factors [30]. Therefore sharing lifestyle/environmental factors might affect the couple's risk for metabolic disorders.

The exact underlying pathophysiology of an association between pregnancy loss and DM and METs is unknown. It is established that gut microbiota triggers metabolic inflammation and subsequent metabolic disorders [57–59]. All the proposed mechanisms were also mentioned as the main players for the occurrence of pregnancy loss [60]. Collectively, sharing common pathways for metabolic dysfunction may provoke the onset of DM or METs in women with history of pregnancy loss. It is worth noting that, recently, Dill-McFarland et al. revealed that cohabiting couples had more similar microbiota composition [61]. In addition, pregnancy loss per se is a traumatic event [62]. Pregnancy loss and following disenfranchised grief negatively impact parents’ health; this also may be prolonged and jeopardize the mental health of couples [63, 64]. The mechanism which links depression and METs is mainly related to low-grade chronic inflammatory conditions [65]. Apart from this, individuals with a history of psychological disorders are more prone to unhealthy behaviors [65]. Recent evidence highlighted inflammatory pathways' role in the pathophysiology of DM and METs [66–68]. It is proposed that paternal lifestyle factors in the preconception period per se are associated with the risk of pregnancy loss [6, 7]. Fossé et al. (2020), in their recent meta-analysis, concluded that paternal smoking > 10 cigarettes per day in the preconception period is linked with an increased risk of pregnancy loss [7]. It is also reported that paternal obesity could affect pregnancy outcomes [8, 9]. Moreover, paternal unhealthy diet may adversely affect pregnancy outcomes [10]. These situations could also induce pro-inflammatory pathways in the exposed men [10]. As a result, a paternal unhealthy lifestyle may directly cause inflammation in exposed men and indirectly associated with an increase in pregnancy loss in their spouses with subsequent further rising metabolic disorder risk.

The findings of this study should be interpreted in the context of weakness and strength. This study was conducted on a longitudinal cohort study with a large number of participants that were followed on average for 15 years. All variables were measured based on a standard protocol with several follow-up visits with 3-years intervals. We adjusted the results based on most potential confounders. Our study has several limitations as well. There is a number of biases concerning study design in terms of indication bias. This is a cohort study which was conducted on an urban population, so the observed effect may be exaggerated, and findings from the research population may not apply to the rural population. The visit intervals were 3-years, and we could not capture shorter variability estimates of metabolic conditions. The length of the time after losing a pregnancy may affect the outcome; since we have no data on the exact date, we assumed the outcomes occurred in the mid-time interval. Diet and nutrition status might influence the metabolic profile, which was not considered in the present study. Moreover, in our study, data related to physical activity were drawn from a questionnaire, which tends to be overestimated by individuals (social desirability bias). In this study, we have no data on the genetic background of participants, which may affect the risk of metabolic abnormality. There was no data on lifestyle and psychological situation during and before pregnancy as potential influential factors.

## Conclusion

Although pregnancy loss is a female-specific factor, may foreshadow the subsequent METs, our study identified a higher risk of subsequent pre-DM and METs in males with a history of pregnancy loss in their spouses. Pregnancy loss could be considered as a future risk factor for metabolic disorders in couples. Despite well-documented impact of some pregnancy complications in developing chronic disease in later life, long-term preventive care for those couples with such history of adverse pregnancy outcomes is lacking [69]. This is the first study that explored the risk of subsequent metabolic disturbances in couples with history of pregnancy loss, more investigations are highly needed to confirm these findings.

## Abbreviations

PL: Pregnancy loss
pre-DM: Prediabetes
DM: Diabetes mellitus
METs: Metabolic syndrome
WHtR: Waist-to-height ratio
BMI: Body mass index
FBS: Fasting blood glucose
SBP: Systolic blood pressure
DBP: Diastolic blood pressure
TG: Triglyceride
LDL: Low-density lipoprotein cholesterol
HDL: High-density lipoprotein cholesterol
TC: Total cholesterol
